# Inverted Papilloma in the Sphenoethmoidal Recess

**DOI:** 10.1016/S1808-8694(15)30767-9

**Published:** 2015-10-19

**Authors:** Eduardo Macoto Kosugi, Rodrigo de Paula Santos, Fernando Freitas Ganança, Rodrigo de Paiva Tangerina, Vinícius Magalhães Suguri, Wellington Yugo Yamaoka, Luis Carlos Gregório

**Affiliations:** 1 Graduate student – MSc – Otolaryngologist; 2 Graduate Student – Doctorate, head of the Rhinology Department – UNIFESP-EPM; 3 Phd in Medicine – UNIFESP – EPM, Affiliated Professor of Neurotology – UNIFESP-EPM; 4 Graduate student – MSc – Otolaryngologist; 5 Graduate student – MSc – Otolaryngologist; 6 Graduate student – MSc – Otolaryngologist; 7 in Medicine – UNIFESP – EPM, Adjunct Professor of Otolaryngology – UNIFESP-EPM. Setor de Rinologia da Disciplina de Otorrinolaringologia do Departamento de ORL-CCP Universidade Federal de São Paulo – Escola Paulista de Medicina

**Keywords:** nose neoplasms, inverted papilloma, sphenoid sinus

## Abstract

Inverted papilloma is a nasal benign tumor that usually arises from the lateral nasal wall, especially from the middle meatus. It has high local invasive likelihood, high recurrence rates and malignancy potential. Sphenoethmoidal recess involvement is rare and is usually due to sphenoid sinus primary disease. In the literature, no case of isolated sphenoethmoidal recess inverted papilloma has been reported yet. The present report describes an exceptional location of inverted papilloma, arising from the sphenoethmoid recess, without involving the sphenoid sinus.

## INTRODUCTION

Inverted papilloma is a rare benign neoplasia that, most of the times, originates from the lateral nasal cavity wall, more precisely in the middle meatus region^1^. Inverted papilloma is also known as fibromyxoid papilloma, transitional cells papilloma, Ewing papilloma, Schneiderian papilloma or Ringertz's papilloma^2^. It represents from 0.5 to 4% of all nasal cavity tumors^2^ and is 25 times less frequent than nasal polyps2. This lesion has an endophytic growth pattern, with epithelium surface inversion to inside the stroma^3^ and, although benign, it is locally invasive and tends towards malignant transformation^2^.

Allergic rhinitis, viral infections, chronic inflammation and environmental factors have been suggested as possible causes for inverted papillomas; however its etiology is still unclear. The typical unilateral presentation is associated with allergic factors or exposure to environmental factors. Viral infection is considered because of its capacity to induce papillomas in other parts of the body; however, the fact that viral infections are much more frequent in children and the very rarity of papillomas in children, tend to discard this theory^4^.

Inverted papillomas typically affect men between 40 and 70 years of age, at the rate of 4:1, and it is rare during childhood and adolescence^4^. It is usually located on the lateral nasal wall, especially in the middle meatus. In a decreasing order of involvement, the paranasal sinuses affected are: maxillary, ethmoidal, frontal and sphenoidal^5^. The exclusive involvement of the sphenoid sinus is rare and its symptoms are not specific1-^3^^,^^6^^,^^7^.

An inverted papilloma in the sphenoethmoidal recess was only described as an extension of the primary disease in the sphenoid sinus^1^, ^2^, ^3^^,^^8^. When there are polyps located in the sphenoethmoidal sinus, it is highly suggestive of sphenoidal sinus involvment^8^. Our goal with the present investigation is to present a case of inverted papilloma involving the sphenoethmoidal recess only, without involving the sphenoid sinus.

## CASE REPORT

**Figure 1:**
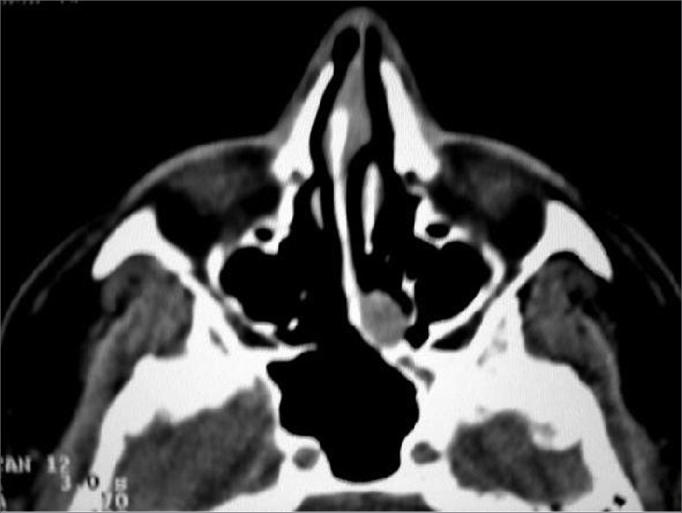
Preoperative CT scan: mucosal thickening in the sphenoethmoidal recess matching the endoscopic finding.

**Figure 2:**
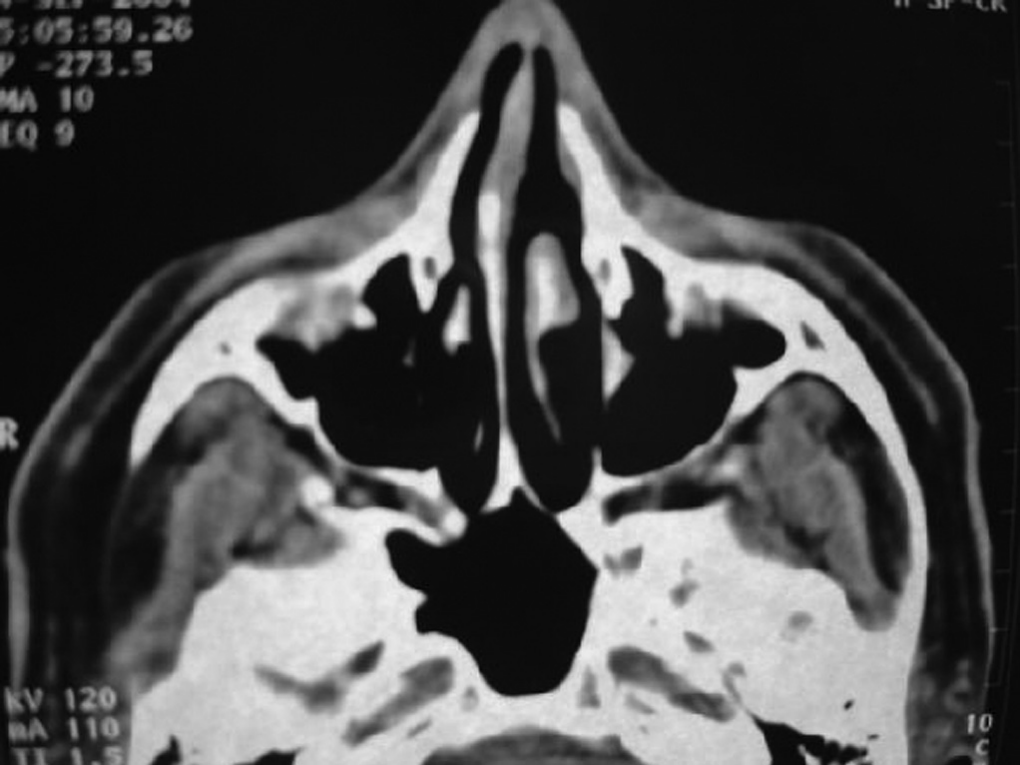
Postoperative CT: complete removal of the lesion.

**Figure 3:**
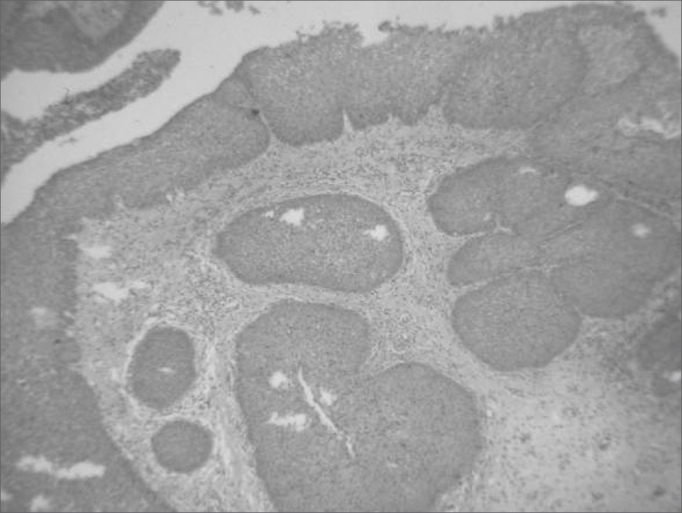
Histology cross-section of the lesion, showing the characteristic inversion of the epithelium surface to inside the stroma of the inverted papilloma.

**Figure 4:**
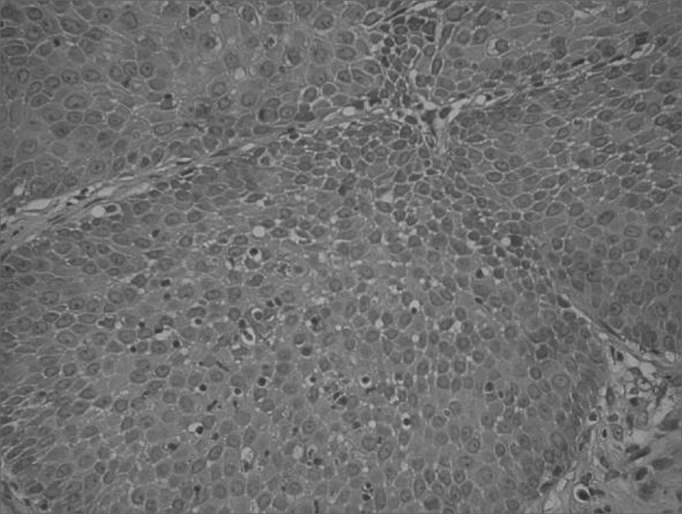
Histology cross-section of the inverted papilloma, without malignancy.

R.G., 40 years old, Caucasian, male, married, business administrator, born and raised in São Paulo – SP. Came to see the doctor about a progressive left side nasal obstruction for 3 months, without improving or worsening. He did not complain of nasal dripping, cough, hyposmia, cacosmia, headache or facial pain. He did not have other complaints. Anterior rhinoscopy was clear. Nasal endoscopy showed a polypoid lesion in his left nasal cavity, coming from the sphenoethmoidal recess, extending to the choana. Paranasal sinus CT scan showed a mass in the sphenoethmoidal recess, of soft tissue density, matching the endoscopy result (Figure 1). The patient was submitted to nasosinusal endoscopic surgery, with prior frozen biopsy. The frozen section showed it to be a papilloma. We then decided for a total sphenoethomoidectomy, and lesion exeresis in its origin in the sphenoethmoidal recess with free mucosal margin. (Figure 2). The sphenoid sinus was not involved. The middle turbinate was normal, and was spared. The pathology exam showed an inverted papilloma, with no malignancy signs (Figure 3, Figure 4). He has been in postoperative follow up for 10 months now, with no signs of recurrence.

## DISCUSSION

Inverted papilloma (just like nasal polyps in general) is more frequently originated from the middle meatus region^7^, ^8^, ^9^, ^10^. By extension, the tumor may involve the adjacent paranasal sinuses, usually involving the ethmoidal labyrinth and the maxillary antrum; however, it can reach the frontal sinus, sphenoid and even the orbit in more advanced cases^1^. Hyams^11^ presents the following incidences in terms of origin: maxillary sinus in 64% of the cases, ethmoidal in 25%, frontal in 8% and sphenoid in 4%. Waitz e Wigand^12^ report the involvement of some paranasal sinus in 88% of the cases, and the anterior ethmoidal was the most frequent with 71% and the sphenoidal, the least frequent, with 10%. Many authors report the sphenoid sinus as the least frequently affected by the inverted papilloma, with very few reports of involvement of this sinus alone^1^, ^2^, ^3^, ^4^^,^^6^^,^^7^.

Nasal polyps originating from the sphenoethmoidal recess are rare^8^. Sethi^8^ considers that a polyp in the sphenoethmoidal recess may strongly suggest pre-existent disease in the sphenoethmoidal sinus, because in their series, only 1 patient (17%) with a polyp in the sphenoethmoidal recess did not have disease in the sphenoid sinus, and this was an inflammatory polyp, not neoplastic, as is the one we are reporting. The few reports of sphenoethmoidal recess involvement by inverted papilloma are, in fact, tumors of the sphenoid sinus, which progressed towards the sphenoethmoidal recess^1^^,^^3^^,^^7^^,^^8^. In the literature there is no report of an inverted papilloma affecting the sphenoethmoidal recess. Most of the times, the inverted papilloma cause unilateral nasal obstruction followed by epistaxis^1^. Nasal obstruction is much more common in cases of disease originating in the middle meatus, being less frequent in the sphenoid sinus^1^^,^^7^^,^^8^. In theses cases, the disease tends to be more insidious and inespecific, more associated with the vulnerable nerve-vessel bundles associated with this sinus^13^. Many authors believe that if we have the involvement of the sphenoid sinus alone, the most common symptoms are headaches and visual disorders^14^^,15^. However, the simultaneous involvement of the sphenoethmoidal recess tends to produce a decompressive effect on the noble structures in contact with the sphenoid sinus^8^. Regarding our patient in this case the papilloma was present in the sphenoethmoidal recess only, without involving the sphenoid sinus; thus, not causing the symptoms stemming from the compression of the vessel-nerve bundles in this sinus. Choanal masses, such as the inverted papilloma, like the case hereby presented, tend to cause nasal obstruction, and symptoms matching those of rhinosinusitis, reaching rare manifestations of auditory symptoms associated with Eustachian tube dysfunction.^8^

More advanced image methods, such as CT scan and MRI may contribute to an increase in the number of cases diagnosed in regions such as the sphenoethmoidal recess and sphenoid sinus^2^. Prior evaluation with nasal endoscopy and CT scan are paramount in order to manage patients with polyps in the sphenoethmoidal recess^7^^,^^8^. Nasal endoscopy guarantees good visualization of the sphenoethmoidal recess and allows us to do biopsy under local anesthesia^8^. In our patient we decided to do a frozen biopsy during the surgical procedure. CT scan provides a good nasal and sinuses evaluation, especially in regards of bone limits. MRI can be necessary to assess invasions of the central nervous system and orbit. Although benign, inverted papillomas have a locally invasive behavior, with about 70% of the cases showing areas of erosion in the CT scan. However, such finding is usually secondary to bone remodeling because of tumor pressure and not because of bone invasion itself^3^.

In terms of histopathology, the inverted papilloma has the characteristic inversion of the epithelial surface to inside the stroma^3^. Prior histopathology evaluation is important, because in inflammatory polyps of the sphenoethmoidal recess all it takes is its excision, while in inverted papillomas, there is the need for a more aggressive approach^7^^,^^8^, because it is associated with malignant transformation in 7 to 15% of the cases^3^. And moreover, inverted papillomas have high and varied rates of recurrence, between 14 and 71%, depending on follow up and the approach used^3^. Because of its high recurrence rate and the potential for malignant transformation, lateral rhinotomy with medial maxillectomy have been considered the best approach3. However, today many authors have reported good results in the surgical management of inverted papillomas by means of endoscopic nasosinusal surgery^1^^,^^3^^,^^7^^,^^8^^,^^12^. We believe the essential point is complete tumor removal, regardless of the pathway used. Obviously, we must use open approaches when the endoscopic one is not enough for a complete removal. The choice between one or the other technique depends on tumor size and location, as well as surgeon's experience^3^. Moreover, a strict follow up must be carried out for at least 3 years, in order to detect recurrences early on^1^.

## FINAL COMMENTS

Inverted papillomas represent a rare disease and may manifest itself in unusual ways, such as in the sphenoidal recess. Nasal endoscopy, image exams and biopsy are necessary for diagnostic assessment and treatment planning. Complete removal must be the crucial concern in order to avoid recurrences. Because of its varied clinical manifestations, this diagnostic must always be considered in cases of unilateral nasal polyps, regardless of its location.
